# The Mystery of Homochirality on Earth

**DOI:** 10.3390/life14030341

**Published:** 2024-03-06

**Authors:** Michael G. Weller

**Affiliations:** Federal Institute for Materials Research and Testing (BAM), Richard-Willstätter-Str. 11, 12489 Berlin, Germany; michael.weller@bam.de

**Keywords:** chemical evolution, enantiomeric excess, chirality, racemate, folding chirality, self-assembly, self-replication, single molecule level, life on earth, prebiotic chemistry, protein folding, conformation, segregation, compartmentalization, nanopore, porous rock, digital immunoassay

## Abstract

Homochirality is an obvious feature of life on Earth. On the other hand, extraterrestrial samples contain largely racemic compounds. The same is true for any common organic synthesis. Therefore, it has been a perplexing puzzle for decades how these racemates could have formed enantiomerically enriched fractions as a basis for the origin of homochiral life forms. Numerous hypotheses have been put forward as to how preferentially homochiral molecules could have formed and accumulated on Earth. In this article, it is shown that homochirality of the abiotic organic pool at the time of formation of the first self-replicating molecules is not necessary and not even probable. It is proposed to abandon the notion of a molecular ensemble and to focus on the level of individual molecules. Although the formation of the first self-replicating, most likely homochiral molecule, is a seemingly improbable event, on a closer look, it is almost inevitable that some homochiral molecules have formed simply on a statistical basis. In this case, the non-selective leap to homochirality would be one of the first steps in chemical evolution directly out of a racemic “ocean”. Moreover, most studies focus on the chirality of the primordial monomers with respect to an asymmetric carbon atom. However, any polymer with a minimal size that allows folding to a secondary structure would spontaneously lead to asymmetric higher structures (conformations). Most of the functions of these polymers would be influenced by this inherently asymmetric folding. Furthermore, a concept of physical compartmentalization based on rock nanopores in analogy to nanocavities of digital immunoassays is introduced to suggest that complex cell walls or membranes were also not required for the first steps of chemical evolution. To summarize, simple and universal mechanisms may have led to homochiral self-replicating systems in the context of chemical evolution. A homochiral monomer pool is deemed unnecessary and probably never existed on primordial Earth.

## 1. Introduction

The preferential occurrence of enantiomers in living systems, known as homochirality, has been regarded as a particularly puzzling phenomenon for decades. Considerable efforts have been and are being made to explain the formation or enrichment of homochiral organic substances on primordial Earth by abiotic processes. It is also unclear whether there is a reason for the emergence of certain enantiomers or whether this selection was random. In the case of amino acids, it is striking that only one enantiomer occurs in proteins. The same applies to nucleic acids and sugars. There are also many examples in the field of smell and taste where different enantiomers cause different sensory impressions. As enantiomers behave identically in chemical and physical terms, there is no obvious reason why the other enantiomer could not also take on the same function. Most chemical syntheses produce racemates, which means the 1:1 mixture of both enantiomers. The issue of chirality is particularly relevant in pharmacy, where the use of racemates as drugs caused severe side effects in some cases [1] and it can be assumed that, as a rule, only one enantiomer/diastereomer has the desired effect in drugs with one or more chirality centers and the other isomers either only represent ballast, which may increase the number and severity of side effects [1,2,3]. Chiral resolution and enantioselective synthesis are therefore one of the most important issues in the development of chemically synthesized drugs [4,5,6].

In contrast to the experience in chemical synthesis that chiral substances are not formed without effort, biological systems are highly enantioselective and often cannot make use of the “wrong” enantiomer. However, it must be distinguished between chirality of small molecules exemplified by drugs or amino acids with asymmetric carbon atoms (enantiomers), and asymmetric conformations of larger molecules, such as proteins. Stable secondary structures, such as alpha-helices or beta-sheets and thus the formation of stable functionalities are significantly facilitated in homochiral polymers [7], although some exceptions are known [8]. Also, in the context of any complex binding pocket of an enzyme or antibody, an enantiomer is not similar to its mirror image, as suggested by a 2D chemical structural formula. Enzymes, receptors, or antibodies usually show a high degree of selectivity towards enantiomer pairs. Karl Landsteiner was one of the first who recognized that antibodies against optically active compounds [9,10] are usually enantioselective. Potential applications of enantioselective antibodies have been described by Hofstetter & Hofstetter [11]. From a biological view, it would be very unfavorable for an organism to develop dual metabolic systems unless there is a strong evolutionary pressure. Assuming a broad mixture of abiotic amino acids as starting monomers or enzyme substrates, it would be much easier for a primordial organism to acquire homologous amino acids of one chirality, than the enantiomers.

In this paper, it is proposed that mechanisms based on abiotic self-assembly led to self-replicating molecules, which inevitably have the tendency to show some preference to one enantiomer.

Looking at the history of chirality as a concept, a similar effect was already apparent in Pasteur’s first report from 1848 [12], in which the spontaneous occurrence of homochirality of tartaric salts in separate crystals was reported. No template or other chiral reagent is required for this process. Looking at the whole batch of salts, the racemate was not altered at all. However, at the microscopic level, the enantiomers separated spontaneously due to preferential crystallization [13]. Considering the first step of crystal formation, two molecules of the same chirality combined and began crystallization of an enantiomerically pure crystal. Unfortunately, many earlier and later papers took the vitalist viewpoint, advocated by Berzelius [14], that some kind of magic “vital force” occurs in living organisms to produce organic and particularly enantiopure compounds.

## 2. Sources of Abiotic Organic Material in the Universe and on Prebiotic Earth

When we leave terrestrial space, simple gases and inorganic minerals, as known from geology, usually dominate. The search for life has so far focused mainly on the search for liquid water, which most scientists regard as a prerequisite for the emergence and development of life forms. Water has been detected on many planets and moons in our solar system. However, if we go one step further and search for organic substances, there is only relatively sparse information from the extraterrestrial realm. Quite a few theories have been proposed to explain the advent of prebiotic organic material [15].

A special case are so-called tholins, which occur on Saturn’s moon Titan and some comets, among others. Tholins are reddish-brown polymers that presumably formed from simple hydrocarbons and nitrogen. Their structure has not been clarified in detail. The hydrolysis of a tholin surrogate lead to a large number of racemic amino acids [16]. Hence, tholins are thought to have potentially played a role in the origin of life on Earth.

Furthermore, meteorites have been shown to be a very valuable source of extraterrestrial organic material [17]. Although the majority of common meteorites consist of metal or rock, a small proportion of meteorites also contain organic material. The best-known meteorite of this type is the Murchison meteorite [18,19,20], which fell in Australia in 1969. Around 100 kg of this material has been collected so far. It belongs to the class of carbonaceous chondrites, which make up around 2–3% of all meteorites. They are also divided into numerous other subclasses. Important in this context is the fact that all meteorites can also be contaminated by terrestrial material and therefore special precautions are necessary when analyzing them. Particularly relevant is the regular occurrence of amino acids, which can undoubtedly be described as substances that can be linked to the origin of life on earth. In the Murchison meteorite, the proteinogenic amino acids glycine, alanine, valine, proline and glutamic acid, and other amino acids that are not normally found in proteins, were detected. The total number of amino acids identified is around 100. As one of the abiotic origins of the amino acids is assumed to be via Strecker amino acid synthesis [21], it is not surprising that racemates are predominantly found. It is also interesting to note that the composition of the amino acids can vary greatly from meteorite to meteorite, so that different formation pathways are postulated. The question of the enantiomer ratios of l- and d-amino acids in meteorites is a controversial topic. Usually only small deviations from the racemic ratio (1:1) are found, which can be caused either by terrestrial contamination or measurement uncertainties. In contrast to meteorites of macroscopic size, which are rarely found and even more rarely belong to the interesting class of carbonaceous chondrites, Earth is continuously hit by a stream of micrometeorites. Estimates assume a mass of 20,000 tons per year [22].

According to a theory by Oparin and Haldane, a prebiotic or primordial soup existed on the early Earth, which could have formed the basis of chemical evolution. In elegant experiments, Miller [23] & Urey [24] attempted to imitate the early Earth’s atmosphere. From simple starting materials such as methane, hydrogen, carbon monoxide, ammonia and water, considerable quantities of organic compounds were formed with astonishing yields under the influence of an energy source, for example, an electric arc. Some proteinogenic amino acids were also formed in this way. In addition, peptides seem to have formed. However, it is disputed whether suitable conditions ever prevailed on Earth. Physical evidence of these products has not yet been found. Therefore, the “primordial soup” has so far remained a laboratory experiment. What is important, however, is the fact that racemates of amino acids are usually formed in this way. And to put it positively: Although the real conditions on early Earth are still unknown, prebiotic soup experiments under different conditions [19,25] led to the formation of amino acids and other organic compounds, which seems to indicate that this mechanism is quite robust. Although some nucleobases have been produced this way, it can be noted that ribose was not found in these experiments and hence the spontaneous formation of a polymer such as RNA is difficult to imagine, whereas the formation of peptide bonds by condensation is much more straightforward.

Finally, theories based on black or white smokers (submarine hydrothermal vents) are still actively followed as places, where the first steps of chemical evolution might have occurred [26]. In this context, it was discussed and examined with real samples from such sites, whether the production of abiotic organic material (e.g., amino acids [27,28] and peptides [29]) might still proceed. However, most results led to the conclusion that organic material is produced by living organisms [30] in these very special biotopes and no indications of recent production of abiotic organic material could be found.

## 3. Amino Acids, Peptides and Proteinoids

### 3.1. Amino Acids or Nucleotides?

Based on the analysis results of extraterrestrial material, the frequent occurrence and diversity of amino acids is astonishing. Since no other complex organic substance group was conclusively found in significant amounts, it can be assumed that amino acids have been the basis of the first phase of chemical evolution. The competing hypothesis of an RNA world [31] requires complex synthesis sequences under special laboratory conditions [32,33] and unclear mechanisms leading to the polymerization of nucleotides. As far as I am aware, only traces of nucleobases [34], but no nucleotides or nucleosides have been detected in extraterrestrial material. Hence, RNA is not the most likely form of self-replicating systems in the very first steps of chemical evolution, just due to a lack of precursors. Instead, it is assumed here that the condensation of amino acids and the subsequent autocatalytic formation of peptides might be the very first molecule on Earth with the ability of self-replication. Although the experimental proof of such a step is lacking, some papers have been published already, which in their overall view, makes this path look realistic.

### 3.2. Condensation of Amino Acids

One of the strongest arguments for the abiotic formation peptides is the simplicity of their formation, only requiring one chemical step, known as condensation. Many different reaction conditions, catalytic and non-catalytic, are known for the formation of peptides from amino acids. First of all, the condensation of amino acids by thermal treatment [35,36,37], which after the loss of water leads to peptide or “proteinoid” [38] formation. The thermal condensation path was advocated and examined by Sidney W. Fox et al. [39]. It was also found that proteinoids are non-random polymers [40]. Without catalysts, relatively high temperatures are often required, which, however, are not unlikely in the context of volcanic activity. In a study of Rohlfing, it could be shown that even temperatures well below 100 °C are sufficient to obtain proteinoids from amino acid mixtures [41]. Recently, it was reported that borate has a significant catalytic activity for the condensation of peptides (up to Gly_39_) under relatively mild conditions [42]. Other publications showed the abiotic formation of peptides under hydrothermal conditions under high pressure [43], although others found it essentially impossible that longer peptides could be formed this way [44]. Commeyras et al. presented an experimental study of a peptide synthesis via N-carboxyanhydrides (NCA) formed by nitric oxides [45]. Finally, the catalytic role of clays, sulfide surfaces and other minerals for the polymerization of amino acids were discussed intensively [46,47].

Without going into the subject of abiotic condensation in more detail, it can be stated that the reactions are relatively straightforward and potentially compatible with the conditions on the primordial Earth.

### 3.3. Formation of Autocatalytic Peptide Oligomers

To enable some information transfer during a potential self-replication, a peptide formed by condensation needs to autocatalyze [48] the formation of the same or a complementary peptide, also known as template replication. Several studies have been published based on the well-known structure element of a superhelical coiled coil. These two intertwined α-helices are stabilized by hydrophobic interactions. The autocatalytic mechanism is not very convincingly shown, yet. However, some ligations of peptides [49,50] have been experimentally shown. Sequence selective sorting of heterotrimers have been shown for collagen-like peptides [51,52]. This shows that peptides can have very distinct self-organization properties. Nevertheless, it should be considered that these first autocatalytic systems had plenty of time and material available. This means that today’s reaction speed considerations cannot be applied here. This is also relevant for any mechanisms requiring amino acid racemization [53] in the formed peptide. Even very low isomerization rate constants could finally lead to homochiral polymers from a racemic pool. Recently, other mechanisms such as amyloid-templating, were proposed [54].

### 3.4. Homochirality in Abiotic Peptides

Obviously, no abiotically formed peptides have been found yet. However, the first very short peptides on primordial Earth might have consisted of a racemic mixture of the amino acids available. There is no solid evidence to suggest otherwise. Recently, an article discussed the topic how homochiral peptides might be formed from racemic mixtures based on the definition of the right location and some thermodynamic considerations [55]. Unfortunately, very few experimental data about chiral selectivity of peptide synthesis have been presented. However, most studies show that homochiral peptides are required to form ordered secondary structures, such as α-helices, although other secondary structures might be possible [7,8,56]. These results seem to support the notion that a specific secondary structure, which has been formed, leads to the preference of one or the other enantiomer for further peptide growth.

## 4. The Mystery of Homochirality

In many papers, homochirality on Earth is seen as a puzzling situation, which requires some explanation [57,58,59,60,61,62,63,64]. Particularly, chemical evolution, irrespective the molecular basis, was seen as unlikely or inhibited if the enrichment of one type of enantiomer did not occur previously. At least a “symmetry breaking” was considered to be essential. The fact that in extraterrestrial material, mainly racemic compounds have been found, was seen as to be in contradiction with the situation today, where homochiral compounds are dominant. This pre-enrichment of one enantiomer was tried to be explained by different mechanisms:

Meierheinrich et al. proposed an asymmetric UV photodecomposition in space [65]. However, in their experiments. only an enantiomeric excess (ee) of up to 2.6% could be obtained. Very recently, a similar hypothesis was pursued by Bocková et al. to explain a small ee of isoleucine in meteorites [66]. In these experiments also, enantiomeric excesses of only 2.05% were achieved. A comprehensive review article was published [67] in which the many claimed enantiomeric excesses in extraterrestrial material were discussed in detail. The overall view seems to be contradictory and did not allow to offer a consistent hypothesis. Nevertheless, the authors stressed the importance of enantiomeric excesses for the identification of any forms of life. From an analytical point of view, the uncertainty of enantioselective measurements cannot completely rule out a statistical or even systematic error. Some studies seem to underestimate the uncertainties of such an analytical undertaking.

In 2009, Noorduin et al. [68] showed a complete deracemization of a slurry of an amino acid derivate in acetonitrile by circularly polarized light. This concept belongs to the most popular ones to explain terrestrial homochirality. Nevertheless, the source of strongly polarized light outside of the laboratory seems to be still unclear. Blackmond [69] discussed the possibility of autocatalysis, which might in fact play an important role. However, in this and other papers, the authors focus on the chirality of small molecules, such as aminonitrile hydration. It seems to be difficult to explain, how this small-molecule autocatalysis might have worked on conditions of the primordial Earth. Another concept is the “Eve crystal”, which was shown with achiral NaClO_3_ [70]. This stunning experiment, probably caused by the crushing of the very first crystal in a solution, is definitely a significant discovery. All experiments based on crystals or crystallization [71] might at some point lead to at least local enantioseparation [72]. On the other hand, a mechanism based on crystallization seems to be not very likely, considering the extremely complex mixtures found in extraterrestrial material or Miller/Urey soups.

Viedma [73] has shown that in the case of mixed crystals in a solution, crystal growth mechanisms might finally lead to a homochiral situation. Very recently, the effect of a magnetic field on the surface of magnetite was used for the enantioselective crystallization of ribo-aminooxazoline. An enantiomeric excess (ee) of about 60% was achieved [74]. Finally, it was postulated that the source of the imbalance of enantiomers is the small difference in energy due to parity violation in the weak force [75,76]. However, the calculated effect was too small to be relevant in this context [77]. Also, spin-polarized leptons (beta irradiation) have been discussed as a source of asymmetry [78].

## 5. The Mystery Disappears

### 5.1. Chirality of Higher Structures

**Figure 1:**
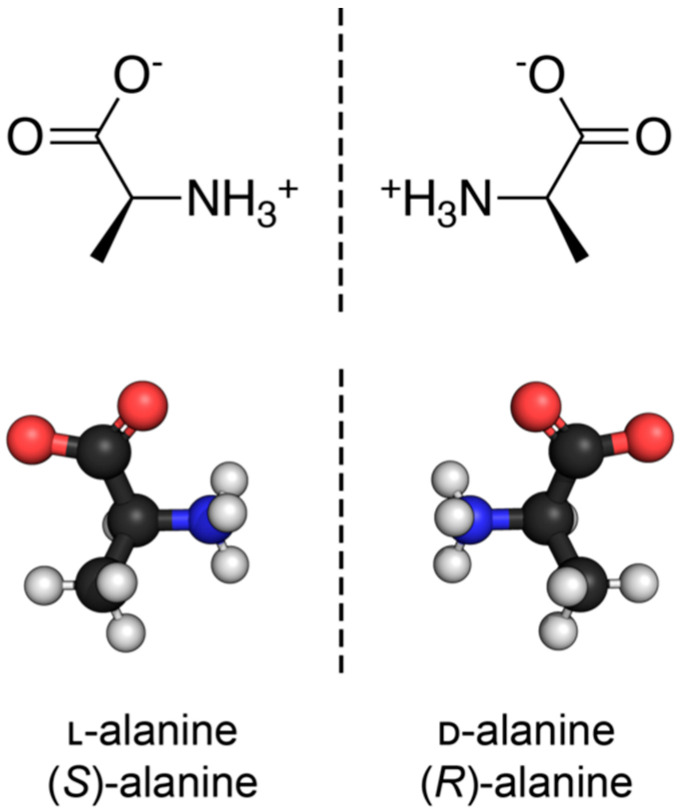
The discussion of homochirality is nearly always focused on asymmetric carbon atoms of monomeric subunits, such as the two enantiomers of the amino acid alanine here. Source: Synpath, Wikimedia, CC-BY-SA-4.0.

**Figure 2:**
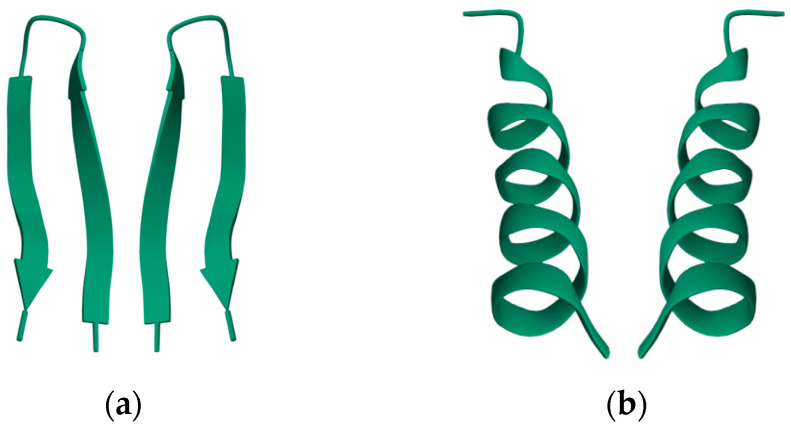
Secondary structures of small peptides with their (non-natural) mirror image. (**a**) A mutant ubiquitin peptide loop (PDB 1E0Q), consisting of 17 amino acids; (**b**) an antimicrobial peptide from *Helicobacter pylori* (PDB 1OT0), consisting of 20 amino acids. The well-formed secondary structures are obviously asymmetric and therefore chiral. Data and visualization are from the RCSB Protein Data Bank (PDB).

**Figure 3:**
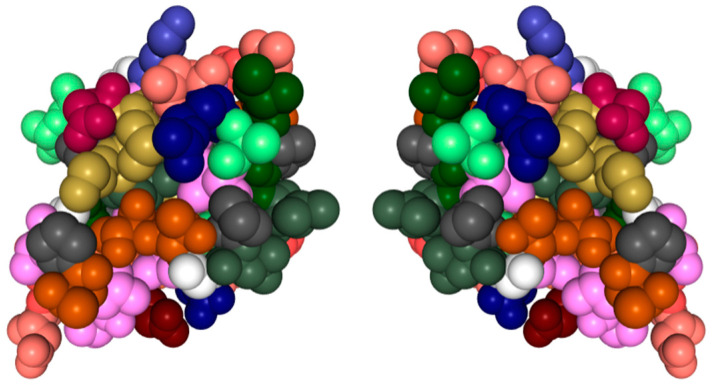
Tertiary structure of a protein with its (virtual) mirror image. (PDB 1KDF): North-Atlantic ocean pout antifreeze protein. Complex structures are essentially always asymmetric. Data and visualization are from the RCSB Protein Data Bank (PDB).

Nearly all discussions around homochirality are limited to the racemic or homochiral property of prebiotic monomers, such as amino acids (Figure 1). Surprisingly, other types, such as axial, helical, planar and conformational chirality [79,80] were often disregarded. For some proteins with crosslinks, the term topological chirality was used [81]. However, it is rarely considered that in any complex molecules, symmetry is highly unlikely, if not impossible in the strict sense. As shown with some real examples, a polymer of growing length will at some point spontaneously form a secondary (Figure 2) and with larger chains a tertiary structure (Figure 3), which always are asymmetric. For this type of chirality, no common nomenclature seems to exist. It might be termed folding chirality.

To illustrate this rule with experimentally determined structures, two examples of relatively short peptides from the Protein Data Bank (PDB) have been selected and shown with their mirror image. In Figure 3 also, an experimentally determined structure of a small antifreeze protein is shown with its virtual mirror molecule. It should be obvious that any of these higher structures are asymmetric.

### 5.2. Statistics of Homochiral Molecules

**Figure 4:**
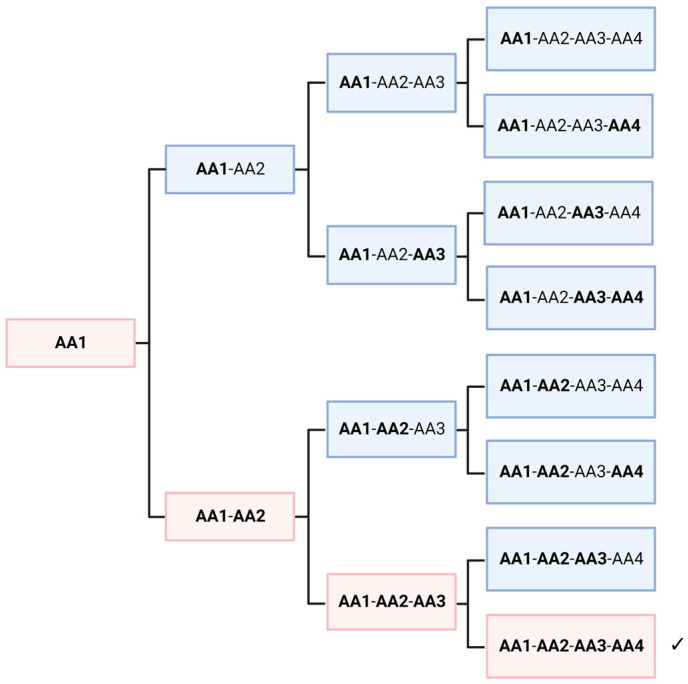
Random polymerization of a racemate always creates a small fraction of a peptide entirely consisting of homochiral monomers (marked by ✓). Regular letters mean one enantiomer, bold ones the other. 1/16 of the product molecules would be homochiral of a selected enantiomeric species. If we assume that the mirror molecule shows chemically the same properties, 1/8 of the oligomers would be homochiral, of one enantiomer or of the other. Created with BioRender.com.

In this thought experiment, we would like to look at the non-selective polymerization of racemic amino acids, regardless of its mechanism. To simplify the situation, we assume only one amino acid, which is present in a racemic mixture. Obviously, a crude mixture of all possible sequences would be formed. As shown in several papers, peptides with amino acids of different chirality fold into different structures [56]. Hence, this mixture of polymers would offer quite dissimilar functionalities. However, it can be shown that, depending on the length of the peptide, some homochiral polymers are always present in low concentrations. In Figure 4, a length of four amino acids is shown, which leads to 12.5% of homochiral polymers. If we assume a length of 30 amino acids for the first self-replicating peptide, a fraction of approximately 10^−9^ would be homochiral. This seems to be a very low yield and perhaps negligible. However, the picture is immediately reversed when we look at the level of individual molecules. A peptide consisting of alanine (Ala_30_) would have a molecular mass of about 2150 g/mol, which is equivalent to 3.58 × 10^−21^ g per molecule. If we assume the fraction above, only about 4 picogram of a random peptide mixture would be necessary to achieve statistically at least one homochiral peptide. Considering geological time and size scales, it is obvious that an astronomically large number of homochiral molecules may have formed on the primordial Earth, without any enrichment or selectivity. Extending this thought experiment to a higher number of amino acids does not significantly change the argument. The probability decreases considerably, but the enormous size of the planet and the millions of years available seem to have compensated for this. On the other hand, several parameters could have increased the probability of the formation on an “active peptide”. Firstly, the length of the peptide might have been relatively short; secondly, a small proportion of the “wrong” enantiomer might not completely abolish the autocatalytic activity; and finally, similar amino acids such as alanine and valine, might be partially interchangeable in their function.

### 5.3. A Digital Assay as a Model for Compartmentalization

Quite a few papers deal with the question of the formation of the first structures, which can be considered to be a primordial cell. Up to now, no convincing hypotheses could be presented. Cell membranes are quite complex partly self-organizing structures. However, for lipids, which would be needed for a bilayer of “modern” structure, no abiotic formation is known. Therefore, it might be sensible to assume that chemical evolution came along without this feature for some time.

**Figure 5:**
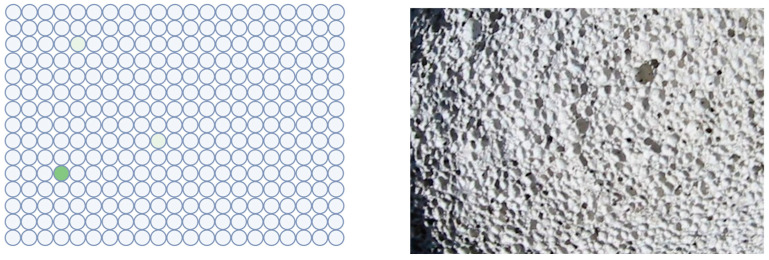
Analogy of digital immunoassay and porous rock. A close-up of a volcanic pumice stone and a schematical result of a digital immunoassay are shown. The enzymatic product (green, fluorescent dye) is kept in a concentrated state by compartmentalization. Without the wells, the dye would be highly diluted and rendered undetectable. Left figure: Created with BioRender.com. Photo source: deltalimatrieste, Wikimedia: “Pomice di veglia.jpg”.

On the other hand, partial compartmentalization seems to be necessary to avoid the dilution of substrates and reaction products to nearly indefinite low concentrations. However, the development of digital immunoassays shows that the confinement of a single catalytic molecule makes a big difference. In bioanalytical chemistry, this approach [82,83,84,85] was an important step for ultimately sensitive assays down to the single molecule level. Their crucial design feature is also compartmentalization. This technology can be realized in different ways; aqueous droplets with reagent beads in “oil” [86], or reagent beads in nanowells made of a solid material [87,88]. The size of the droplets is chosen this way so that each compartment preferentially contains a single enzyme molecule. Caused by the separation of the liquid volumes, any enzymatic product is kept highly localized and is not diluted to undetectable concentration. In Figure 5, a typical result is shown in schematical representation. It should be noted that the pumice stone is only a symbolic representation. The confinement effect is usually stronger in nanosized wells.

Transferred to the situation of chemical evolution, it is possible that simple ways of compartmentalization supported the development of more complex systems. However, not complex bilayers or other cell membranes, but liquid trapped in porous rocks, might have been the “reagent tubes” of chemical evolution. Since such rock pores often are not completely closed, even some slow, perhaps size-dependent material exchange might have occurred to avoid the reactions in the droplets running into an equilibrium and coming to a halt. Furthermore, the surface of the rock might have had some catalytic function. A single self-replicating peptide in such a rock nanopore could have been the first step to life.

## 6. Conclusions

The combined analysis of the collected publications in this review allows some preliminary conclusions, although some research deficits are obvious. This summary is based on some limited material evidence, discussions in the cited papers and derived probabilities. It cannot be ruled out that some completely unknown processes are responsible for the first steps of evolution. Their discovery must be left to future research efforts. Today, the following can be stated:

The homochirality of organic precursors is not a prerequisite for the emergence of life. Therefore, hypotheses for abiotic enrichment or any other enantioselective preference for such monomers are not required. Simple mechanisms are proposed, which all do not need a homochiral abiotic precursor pool.

1. Homochiral polymers can be formed from racemic starting materials at the single-molecule level, which then may have multiplied by autocatalysis. Which enantiomer would have formed the first autocatalytic peptide would be left to chance. If oligomers of different lengths and structures are compared, it would not necessarily be the first molecule that would win the race, but the one that replicated the fastest.
2. All polymers that can form at least secondary structures are asymmetric, regardless of their origin or which monomers they consist of. Even completely achiral monomers would constantly form asymmetric polymer molecules (conformations, folding chirality).
3. Self-replicating molecules would have a very strong tendency to form products of a higher enantiomeric excess, if the “first” self-replicating molecule was not already completely homochiral and inherit these properties to the next generation of molecules, probably due to a better formation of secondary structures and a more efficient mechanism of self-replication.
4. Partial compartmentalization, e.g., in pores of a rock might deliver a suitable environment for even slow autocatalytic processes without excessive dilution over time.

For a long time, the abiotic starting materials (primordial soup of any origin) would have dominated the situation on Earth. Individual, self-replicating molecules could have existed and reproduced as homochiral units in an “ocean” of racemic substrate. It seems likely that the “other” enantiomers first would simply remain but were finally either abiotically [53] or enzymatically racemized until they were all consumed by the self-replicating units. Only after the lack of primordial substrate did homochiral molecules become dominant on Earth through novel biochemical synthesis paths.

Based on repeated evidence from extraterrestrial material, it seems more likely that peptides and not RNA were formed in a first step. It is also possible that the first phase was determined by partially segregated aqueous droplets for example in porous rocks rather than complex cells.

In the context of current space missions to extraterrestrial objects, it makes little sense to search for chiral compounds as primary sources of homochirality on Earth. Even if some enantiomeric excesses would be unequivocally detected, this effect would be canceled out by the much stronger effects discussed here. However, the above conclusions make the search for chiral compounds as indication for possible extraterrestrial life (“biosignatures”) even more relevant, including compounds on a different chemical basis. Homochirality seems to be an inevitable and universal feature of life.

It is also helpful to abandon the notion of the spontaneous emergence of complex ensembles and structures based on homochiral monomers as the beginning of life on Earth. Relatively small, single molecules with the ability to self-replicate by enantioselective autocatalysis could be the most likely first step in chemical evolution. Based on this hypothesis, a pool of homochiral abiotic precursors is unlikely and probably never existed.

## Data Availability

Not applicable.
